# Applications of graph theory to the analysis of fNIRS data in hyperscanning paradigms

**DOI:** 10.3389/fncom.2022.975743

**Published:** 2022-09-14

**Authors:** Amanda Yumi Ambriola Oku, Candida Barreto, Guilherme Bruneri, Guilherme Brockington, Andre Fujita, João Ricardo Sato

**Affiliations:** ^1^Center of Mathematics, Computing and Cognition, Universidade Federal do ABC, São Bernardo do Campo, Brazil; ^2^NRF South Africa Chair: Integrated Studies of Learning Language, Science and Mathematics in the Primary School, University of Johannesburg, Johannesburg, South Africa; ^3^Physics Department, Aeronautics Institute of Technology, São José dos Campos, Brazil; ^4^Center for Natural and Human Sciences, Universidade Federal do ABC, Santo André, Brazil; ^5^Computer Science Department, Institute of Mathematics and Statistics, University of São Paulo, São Paulo, Brazil

**Keywords:** fNIRS, graph theory, degree centrality, eigenvector centrality and modularity, neuroscience

## Abstract

Hyperscanning is a promising tool for investigating the neurobiological underpinning of social interactions and affective bonds. Recently, graph theory measures, such as modularity, have been proposed for estimating the global synchronization between brains. This paper proposes the bootstrap modularity test as a way of determining whether a pair of brains is coactivated. This test is illustrated as a screening tool in an application to fNIRS data collected from the prefrontal cortex and temporoparietal junction of five dyads composed of a teacher and a preschooler while performing an interaction task. In this application, graph hub centrality measures identify that the dyad's synchronization is critically explained by the relation between teacher's language and number processing and the child's phonological processing. The analysis of these metrics may provide further insights into the neurobiological underpinnings of interaction, such as in educational contexts.

## 1. Introduction

Hyperscanning, the recording of brain activity from two or multiple individuals, is a promising tool for investigating the neurobiological underpinning of social interactions and affective bonds. Montague et al. (2002) was the first hyperscanning study. It performed experiments in which participants could interact with each other while functional MRI were acquired in synchrony with the behavioral interactions. The data was recorded through a simple game of deception between pairs of subjects. The asymmetric and asynchronous interactions analyzed in this study during interactions are still used today in studies between two or more individuals.

Since then, hyperscanning studies have shown, for instance, how to predict leaders and followers (Sänger et al., 2013), the influence of social proximity between teacher and student in education (Bevilacqua et al., 2019), and how to detect track engagement and classroom social dynamics (Dikker et al., 2017).

The proposed methods for hyperscanning analysis can be classified into connectivity measures, correlation analysis, information flow analysis, and graph theory measures (Czeszumski et al., 2020). A common trait between connectivity measures, correlation analysis, and information flow is that they determine locally the strength of the synchronization between each pair of inter-brain regions. Although such an analysis is useful, one might also be interested in understanding brain-to-brain synchronization on a global scale. Graph theory provides useful tools for this line of investigation.

A Graph is a mathematical representation of a network and is essentially a list of nodes and list of connections between nodes (Kunegis, 2013). Measures such as graph modularity have been used for estimating the global strength of synchronization of groups of brains. For instance, De Vico Fallani et al. (2010) furthers the understanding between brain networks and neural mechanisms responsible for human social interactions. Also, Liu et al. (2021) shows global estimates of drum-beat synchronization in the team-focus condition of a nine-person drumming task. However, it remains an open question to test whether a group of individuals are globally synchronized or not.

Such a question is often useful. For instance, one might require a screening tool to remove pairs of desynchronized brains from a sample. In this exploratory study, we propose a bootstrap modularity test for determining whether two subjects are coactivated. This method is demonstrated with an application to fNIRS data from a naturalistic hyperscanning experiment in which a teacher presents a mechanism for summing two numbers to a child.

More specifically, we build a graph of inter-brain connections between regions. The proposed bootstrap modularity test is used to screen out not coactivated individuals. Next, we use a combination of graph hub centrality measures to identify which brain regions are most influential in explaining coactivation. We show that, in most pairs, coactivation is mediated by the relation between the teacher's prefrontal cortex and the child's right temporoparietal junction. We seek to provide a novel framework to assess the neural dynamics synchronization across pairs of different brains.

The data used in this work refers to an experimental context involving arithmetic procedure as described in Brockington et al. (2018), Barreto et al. (2021). This task was chosen under the perspective of education as a cooperative social process that occurs at the zone of proximal development, a key construct in Lev Vygotsky's theory of learning and development (McLeod, 2019).

Using the aforementioned data, this paper introduces a new method based on graph theory that explains how the prefrontal cortex region (PFC) connects with the right temporoparietal junction (rTPJ). While the PFC is known to be involved in processes of high order cognition such as counting and calculating (Fuster, 2000; Artemenko et al., 2018; Soltanlou et al., 2018), the temporo-parietal junction (TPJ) is involved in social functions such as empathy and mentalizing (Van Overwalle, 2009; Carter et al., 2012). Thus, we hypothesized that activity between teacher and child would couple in a cross-link.

## 2. Methods

### 2.1. Participants

Five pairs of teacher-child which reported no cognitive disabilities participated in the experiment, as described in Barreto et al. (2021): Five children aged between 3 and 5 (four boys) and four adults aged between 21 and 28 (two males). Children were recruited by advertisements in a public school and teachers were tutors from a Science Museum at the University of São Paulo, Brazil.

We obtained informed and written consent from all adult participants and parents/legal guardians of all non-adult participants. All subjects had normal vision and hearing and no neurological or psychiatric disorders history. The Federal University of ABC—Ethics Committee approved all aspects of our experiment, which was performed following all relevant guidelines and regulations. All subjects participated voluntarily and without any financial compensation.

### 2.2. Experiment

Experimental data was obtained using fNIRS. The fNIRS provides safe, comfortable, and realistic means for data collection in a natural condition. We used safe levels of light (with wavelengths between 650 and 900 nm) to infer the oxygenation variation level of brain tissue in a non-invasive way. The light penetrates the biological tissue and reaches the cortex, allowing the analysis of oxyhemoglobin (O2Hb), deoxyhemoglobin (HHb), and total hemoglobin (tHb; tHb = O2Hb + HHb) from cerebral blood (Delpy and Cope, 1997).

The teacher-student data was collected in a hyperscanning paradigm, as described in Brockington et al. (2018), Barreto et al. (2021).

Briefly, the dyads interacted in a task which the teacher presents the mechanisms to sum two numbers (1 to 12) using matchsticks in a context of a space-race game with the child. They need to move two pawns (representing the child and the teacher) on a pathway board marked with numbers. At first, after throwing two six-sided dice, the player who got the highest sum started the game. They continued the race by walking the steps to the sum of the dice numbers until the finish line. Brockington et al. (2018) presents more details about the experimental design, such as a scheme with the setup of the experiment and characteristics of the collected signals, in the Experiment 1 subsection under the Case Studies section.

### 2.3. Data acquisition and preprocessing

We collected the data as described in Barreto et al. (2021). We used a NIRScout (NIRx Medical Technologies, New York, NY, United States) with a sampling rate of 7.81 Hz, 16 sources and detectors were placed in the prefrontal cortex (PFC) and right temporoparietal junction (rTPJ) as demonstrated in the Supplementary material. We chose those regions because of our interest in social functions such as empathy and mentalizing (Artemenko et al., 2018; Brockington et al., 2018; Barreto et al., 2021).

Raw data were processed using a home-made MATLAB script from our research group, as described in Barreto et al. (2021). We used a 0.01–0.2 Hz bandpass filter to reduce physiological signal artifacts at the cutoff frequencies of the global deviations (<0.01 Hz), cardiac cycles (>0.5 Hz) and systemic interferences such as respiration rate (>0.2 Spearman). Other motion artifacts (spikes) were removed. The modified Beer-Lambert law (Delpy and Cope, 1997) was then applied to calculate concentration changes in oxygenated hemoglobin (HbO_2_) and deoxygenated hemoglobin (HHb). Afterward, we used the mean of the entire timeline as a baseline and differential path length factor (DPF) of 7.25 for the 760 nm and 6.38 for 850 nm wave lengths.

### 2.4. Proposed methodology—Graph measures for intercerebral inferences

Centrality measures identify which nodes are most relevant in the composition of networks and supports to find the main edges (the connection between brains). In the following, we analyze the centrality measures aggregated over all dyads and individually.

We processed all learning algorithms in R version 3.6.3. We illustrate our proposed framework by applying it to the *O*_2_Hb signals from the five teacher-child dyads. The dyad's adjacency matrix, ***A***, was obtained by calculating the Spearman correlation between each of the 18 teacher channels against each of the 18 child channels.

Since we intend to find inter-brain visualization, we zeroed all intrabrain correlations in ***A***. Also, we zeroed out correlations below the delimited *corr* = 0.15 to ensure better visualization. We chose this cut so that at most 10% of the edges were retained in each dyad. Other cutoffs would not change the qualitative nature of the results, but could lead to a more difficult visualization of the graph. The five resulting matrices represent undirected graphs over the dyad's brain regions.

For each graph, we calculated the modularity and centrality measures using the “igraph” (version 1.2.5) library of R (Csardi and Nepusz, 2006), which uses the method proposed by Kleinberg (1999) for calculating the eigenvector centrality from Bonacich (1972).

The modularity, introduced by Newman and Girvan (2004), measures how well we can divide a graph into two or more isolated groups of nodes. In this application, high modularity indicates the lack of relevant interbrain connections. The lower the modularity, the higher the coactivation between two subjects.

Community structure in networks reflects the concentration of edges of a graph within communities compared to the random distribution of connections across all nodes, regardless of communities.

Let **k_i_** and **k_j_** be the degrees of nodes *i* and *j*, respectively, of the graph **G**. Also, let **G_ij_** be 1 if there exists an edge between *i* and *j* and 0, otherwise. Suppose the graph's edges were randomly distributed between each pair of nodes. Then, the expected number of edges between *i* and *j* would be $\frac{{\text{k}}_{\text{i}}{\text{k}}_{\text{j}}}{{\sum_{\text{i}}\text{k}}_{\text{i}}}$.

Thus, the modularity *Q* is given by the sum of the difference:

${\text{G}}_{\text{ij}}\frac{{\text{k}}_{\text{i}}{\text{k}}_{\text{j}}}{{\sum_{\text{i}}\text{k}}_{\text{i}}}$ over all pairs of nodes *i, j* within the same group (Newman, 2006).

Conceptually, modularity evaluates the number of interbrain connections concerning the expected number of edges between the same group of nodes but in a random graph with the same sequence of edges.

**Figure 1:**
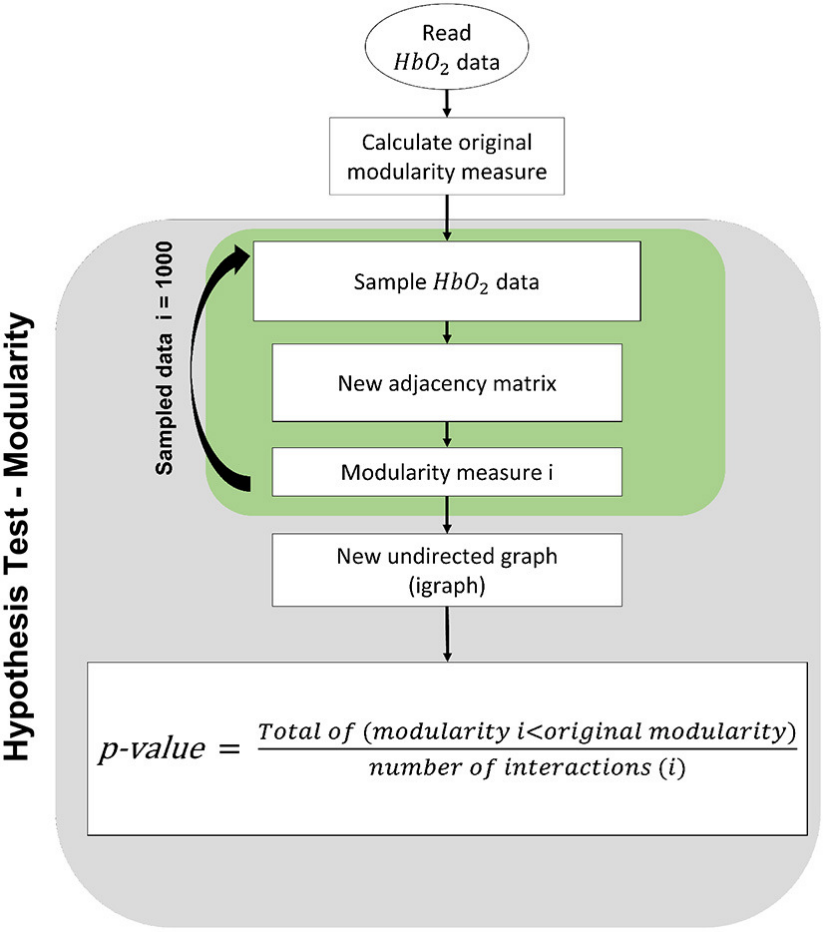
Modularity hypothesis test procedure: For each analyzed dyad, we calculated the consistency of the modularity found. We test the hypothesis that the teacher's channels are independent of the child's channels. We applied a permutation test in which, for each iteration, we permuted the teacher's channels.

In order to test whether the teacher and child are synchronized, we applied a bootstrap hypothesis test. In this test, the null hypothesis is that teacher and child are not synchronized. The modularity is calculated on resampled data in Figure 1 repeatedly. A *p*-value is obtained by counting how often the modularity was higher than in the original data.

In addition to modularities, we seek to identify measures for the level of relevance of nodes present in graphs. Centrality indices are given by a real-valued function on the nodes of a graph, which the values produced are expected to provide a ranking that identifies the most critical nodes in the network.

There are some ways to calculate measures of centrality, which vary according to the relevance index to be studied, such as a node's number of direct connections (degree centrality) or the sum of the centralities of its direct neighbors, named eigenvector centrality (Zhang and Luo, 2017).

We used degree centrality to find the nodes which were generally more connected. By aggregating centrality measures over all obtained graphs, one obtains an overall idea of the central nodes and their communities. We defined the node count as the sum over all graphs of the number of edges connected to a node. Also, we defined the node relevance as the node count divided by the total number of edges over all graphs. That is, the node relevance is the relative frequency of edges connected to a given node over all graphs.

During interactions, the eigenvector centrality measures prominent channels in the brain network hierarchy (Binnewijzend et al., 2014). While degree centrality provides a simple count of the number of connections a node has, eigenvector centralities recognize that not all connections are equal and that some nodes can influence much better than others (Newman, 2008).

Let **A** be the adjacency matrix of a graph **G** with nodes *i* and *j* and denoting by **x_i_** and **x_j_** their respective centralities. Then, the score of the vertex *i* is determined by:


$${\text{x}}_{i}=\frac{1}{\lambda }\overset{n}{\underset{j=1}{\sum }}A_{ij}x_{j},$$

where λ is a constant. By defining the centrality vector **x** = (*x*_1_, *x*_2_, ..., *x*_*n*_) it is possible to rewrite:


λx=Ax

Thus, we obtain **x** an eigenvector of the adjacency matrix with eigenvalue λ.

Eigenvector centrality attributes a value to each fNIRS channel in the brain such that each one receives a large value if it is strongly correlated with many other central nodes within the network. In practice, the eigenvector centrality determines for each channel (vertex) a centrality that depends simultaneously on the number and quality of its intercerebral connections. In other words, a channel with many connections does not necessarily outperform a channel with a smaller number of connections if the quality of the latter relationships is better.

With the centrality measures calculated for each channel analyzed in the dyads, we were able to visualize, per dyads, which channels are considered a *hub* in the influence of other regions.

We calculated the positions of the fNIRS channels based on the 10-10 EEG system from the library “eeg_positions” (Appelhoff, 2021) to improve the graphical visualization. We exported and interpolated the EEG coordinates to fNIRS, which could be generated for any dyads and scaled according to the assembly. More details about the assembly and detailed picture description can be seen in the Supplementary materials.

## 3. Results

This section describes the main methodological results that were obtained. Sections 3.1, 3.2, and 3.3 describe, respectively, the adjacency matrix, modularity measures, and centrality measures.

### 3.1. Adjacency matrix

The proposed graph analysis of interbrain connectivity relies on an adjacency matrix, summarized in Table 1. Most of the teacher's nodes refer to cognitive functions such as task management, planning, working memory, attention, and executive function (Koessler et al., 2009; Bandeira et al., 2019). Areas relevant to phonological processing and emotional responses also were involved. Most of the children's nodes refer to phonological processing, and some to emotional responses and cognitive functions (Zimeo Morais et al., 2018). Table 1 indicates low interbrain correlations in the first dyad.

**Table 1:** The adjacency matrix is composed of Spearman correlations between each of the 18 teacher channels against each of the 18 child channels.

**Correlation**	**Pair 1**	**Pair 2**	**Pair 3**	**Pair 4**	**Pair 5**
<0.1	318	286	273	272	276
0.1–0.15	6	24	26	23	33
0.15–0.2	0	11	18	19	11
0.2–0.25	0	3	4	6	4
		(**FP1-AF7**; **CP4-CP6**)	(P4-P6; AF4-F6)	(**AF3-F5**; **CP4-CP6**)	(**FP2-AF8**; FP1-AF7)
		(**AF3-F5**; TP8-P8)	(AF7-F5; **CP4-CP6**)	(CP6-TP8; **CP6-TP8**)	(C6-CP6; FP1-AF7)
		(P8-P6; C4-CP4)	(CP6-TP8; **CP6-TP8**)	(P8-P6; **C4-C6**)	(**FP2-AF8**; FP2-AF4)
			(**FP2-AF8**; TP8-P8)	(**AF3-F5**; CP4-P4)	(C6-CP6; FP1-AF3)
				(**FP1-AF7**; **CP4-CP6**)
				(**FP2-AF8**; **CP4-CP6**)
0.25-0.3	0	0	3	2	0
			(**FP2-AF8**; **CP4-CP6**)	(P4-P6; **C4-C6**)
			(AF8-F6; **CP6-TP8**)	(C4-CP4; AF4-F6)
			(**FP1-AF7**; **CP4-CP6**)
>0.3	0	0	0	2	0
				(CP4-CP6; **C4-C6**)
				(CP6-P6; **C4-C6**)
Total	324	324	324	324	324

This table counts for each pair of how many intra-brain correlations fall in each interval. The number of inter-brain connections of each pair of graphs is determined by the sum of lines in each interval. Pair 1 has low intra-brain correlations. For intra-brain correlations above 0.2, we identified the teacher-child edges in parenthesis. The number of connections in each pair was considered in the analysis of modularity and in the hypothesis test. The most frequent channels are highlighted in bold.

### 3.2. Modularity measures

Next, we used the bootstrap modularity test to check which pairs were coactivated. As an initial probe, we found out that no pair of children from different trials were coactivated, which proves the robustness of the bootstrap modularity test. Also, Table 1 points to the hypothesis that all teacher-child dyads are both coactivated, except for the first. These hypotheses can be confirmed through the modularity test: only the first dyad had no coactivation (*p* = 0.36). The complete *p*-value results of all pairs can be seen in the Supplementary materials. Based on these results, the centrality hubs for interbrain connectivity were studied only in the four remaining dyads.

### 3.3. Centrality measures

The aggregated centrality measures were obtained through node counts and node relevance, as described in Section 2.4. For better visualization, we present the teacher's and child's node count and relevance in Table 2. The Table 2 shows that the three most relevant channels for the teachers (FP2-AF8, FP1-AF7, and AF3-F5) belong to the PFC. Similarly, the three most relevant channels for the children (CP4-CP6, C4-C6, and CP6-TP8) belong to the rTPJ.

**Table 2:** Teacher's node count and node relevance.

**Teacher**				**Child**			
**Channel**	**Position**	**Node count**	**Node relevance**	**Channel**	**Position**	**Node count**	**Node relevance**
V5	FP2-AF8	5	21	V12	CP4-CP6	7	29
V1	FP1-AF7	3	13	V9	C4-C6	4	17
V4	AF3-F5	3	13	V13	CP6-TP8	3	13
V13	CP6-TP8	2	8	V1	FP1-AF7	2	8
V16	P4-P6	2	8	V8	AF4-F6	2	8
V11	C6-CP6	2	8	V17	TP8-P8	2	8
V18	P8-P6	2	8	V10	C4-CP4	1	4
V12	CP4-CP6	1	4	V15	CP4-P4	1	4
V10	C4-CP4	1	4	V7	FP2-AF4	1	4
V6	AF8-F6	1	4	V3	FP1-AF3	1	4
V14	CP6-P6	1	4	V5	FP2-AF8	0	0
V2	AF7-F5	1	4	V4	AF3-F5	0	0
V9	C4-C6	0	0	V16	P4-P6	0	0
V8	AF4-F6	0	0	V11	C6-CP6	0	0
V17	TP8-P8	0	0	V6	AF8-F6	0	0
V15	CP4-P4	0	0	V14	CP6-P6	0	0
V7	FP2-AF4	0	0	V18	P8-P6	0	0
V3	FP1-AF3	0	0	V2	AF7-F5	0	0

Nodes FP2-AF8, FP1-AF7, and AF3-F5 have the highest node relevance. These nodes belong to PFC. Children's node count and node relevance. Nodes CP4-CP6, C4-C6, and CP6-TP8 have the highest node relevance. These nodes belong to rTPJ.

**Figure 2:**
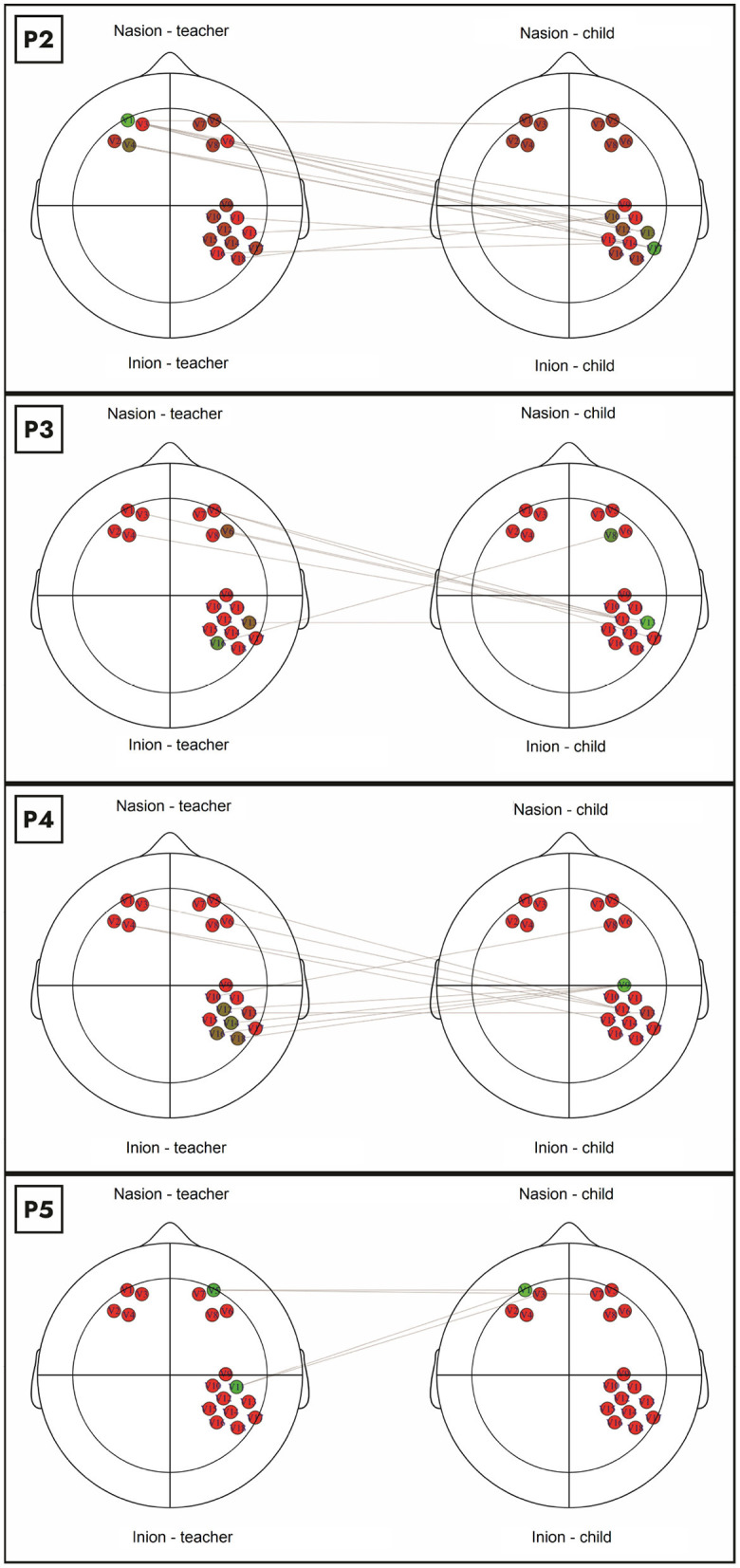
We identified several relationships of the PFC channels (teacher and child) to the rTPJ channels in the four pairs with coactivation. In dyads 2, 3, and 4, the primary interbrain connections occur between the teacher's PFC and the child's rTPJ. This pattern was inverted in dyad 5, where the primary connection is between the teacher's rTPJ and the child's PFC.

We further evaluated this suggestion by inspecting each dyad's graph. Figure 2 show the primary connections between the analyzed channels of all pairs with coactivation. The colors in the graph indicate the eigenvector centrality of each node – from red (low) to green (high). This figure corroborates that, in the dyads 2, 3, and 4, the primary interbrain connections occur between the teacher's PFC and the child's rTPJ. This pattern was inverted in dyad 5, where the primary connection is between the teacher's rTPJ and the child's PFC.

Using centrality measures, we could determine the most relevant cortical regions involved in teacher-child coactivation. The teacher's most relevant nodes relies on the regions related to phonological processing, emotional responses, language and number processing, spatial cognition, memory retrieval, attention, and cognitive functions. The child's most relevant nodes relies on the regions related to task management, planning, attention, and executive function.

## 4. Discussion

The proposed methodology demonstrated the possibility of using graph theory to detect coactivation and identify hub areas involved during the interaction. In this methodology, modularity measures identify the existence of coactivation between brains and centrality measures indicate main brain regions involved in the inter-brain connections. In the featured experiment, our proposal suggests that the teacher's PFC is usually strongly connected to the child's rTPJ.

Our study has some limitations. It involved a relatively small sample of children and teachers with different math achievement levels, representing an explicit limitation in terms of generalizing results. The limited number of sensors restricted the study to analyzing only the rTPJ and prefrontal regions (Barreto et al., 2021), as well as not allowing us to use short distance detectors. Short distance could assist in the exclusion of extracerebral signals around the sources in fNIRS collection data (Tachtsidis and Scholkmann, 2016).

Hyperscanning has successfully been used for establishing the neurobiological underpinning of social interactions and affective bonds (Vanutelli et al., 2017). In particular, hyperscanning analysis provides relevant indicators that can help teachers choose teaching materials, establish and maintain a good teacher-student relationship, and attach importance to the role of interaction in teaching activities (Cui et al., 2012; Dikker et al., 2017; Bevilacqua et al., 2019). While traditional approaches detect local coactivation between inter-brain regions, graph theory provides measures of global coactivation.

Recent theoretical reviews of graph theory measures have noted their potential key application to hyper scanning studies (Czeszumski et al., 2020). However, it remains an important open question to determine whether two brains are synchronized. In this research, we sought to identify connections between the brains of dyads using graphs constructed from the correlation matrix between subjects.

Measures such as modularity bring a new perspective to understanding the neural foundations of dynamic social interactions (Czeszumski et al., 2020). For instance, our study proposed a new method to detect coactivation between dyads, the bootstrap modularity test.

Furthermore, centrality measures can be helpful tools for mapping the global architecture of a brain network (Sporns, 2018). While degree centrality brings insights into the existing connections in the network, the eigenvector centrality measures the transitive influence of nodes (Bonacich, 2007). In our study, degree centrality identified the central nodes involved in teacher-child coactivation. It suggests that the interbrain connections involve mainly the teacher's PFC and the child's rTPJ. A more nuanced analysis was possible by visualizing the eigenvector centralities in each graph. While in dyads 2, 3, and 4, the primary interbrain connection occurred between the teacher's PFC and child's rTPJ, this relation was inverted in dyad 5. Other studies also suggest the existence of a PFC-TPJ interbrain network in other naturalistic or cooperative behaviors (Schurz et al., 2014; Xue et al., 2018; Duan et al., 2020).

Further investigations are necessary given the high level of complexity of naturalistic experiments. For instance, the child's phonological decoding was highly relevant in all coactivated pairs. This fact highlights the importance of future studies that also collect data from the left TPJ. This enhanced data together with cognitive tests for language would provide a better understanding of social interactions in children aged 3–5 years.

## Data availability statement

The raw data supporting the conclusions of this article will be made available by the authors, without undue reservation.

## Ethics statement

The studies involving human participants were reviewed and approved by Federal University of ABC-Ethics Committee (CAAE 41837515.2.0000.5594). Written informed consent to participate in this study was provided by the participants' legal guardian/next of kin.

## Author contributions

CB, GBru, GBro, and JS collected the data. AO and JS analyzed the data and wrote the manuscript. CB, AF, and JS revised and contributed to improving the quality of the manuscript. All authors have read and agreed to the published version of the manuscript.

## Funding

The authors are thankful to São Paulo Research Foundation (FAPESP grant numbers 2018/04654-9, 2018/21934-5, and 2019/17907-5).

## Conflict of interest

The authors declare that the research was conducted in the absence of any commercial or financial relationships that could be construed as a potential conflict of interest.

## Publisher's note

All claims expressed in this article are solely those of the authors and do not necessarily represent those of their affiliated organizations, or those of the publisher, the editors and the reviewers. Any product that may be evaluated in this article, or claim that may be made by its manufacturer, is not guaranteed or endorsed by the publisher.
