# Supplementary figures and images for: Applications of graph theory to the analysis of fNIRS data in hyperscanning paradigms
Source: Front Comput Neurosci. 2022 Sep 14;16:975743. doi: 10.3389/fncom.2022.975743 (PMC9521601; doi:10.3389/fncom.2022.975743)

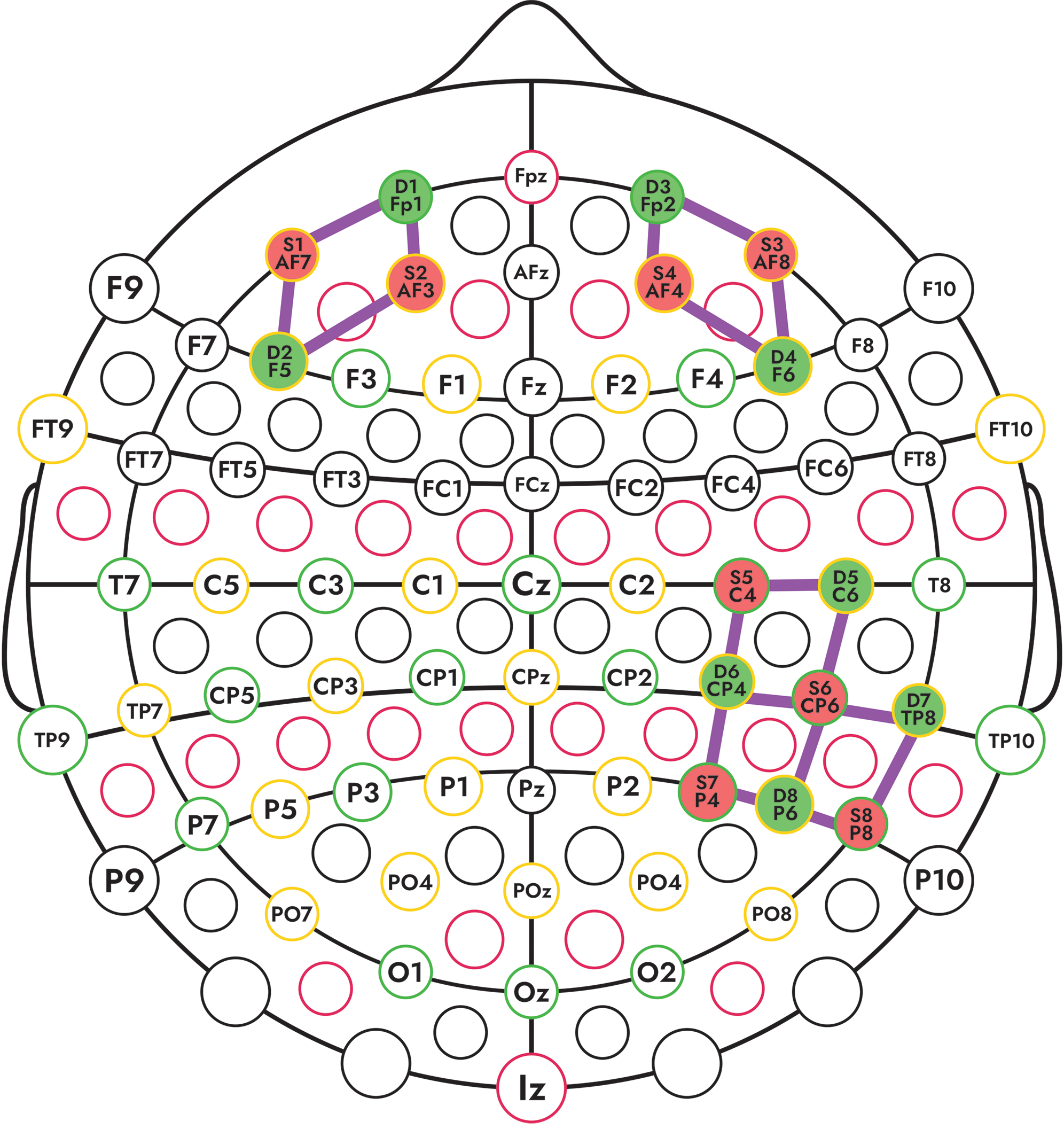

Supplement: Supplementary file 1 [file Image_1.TIF]

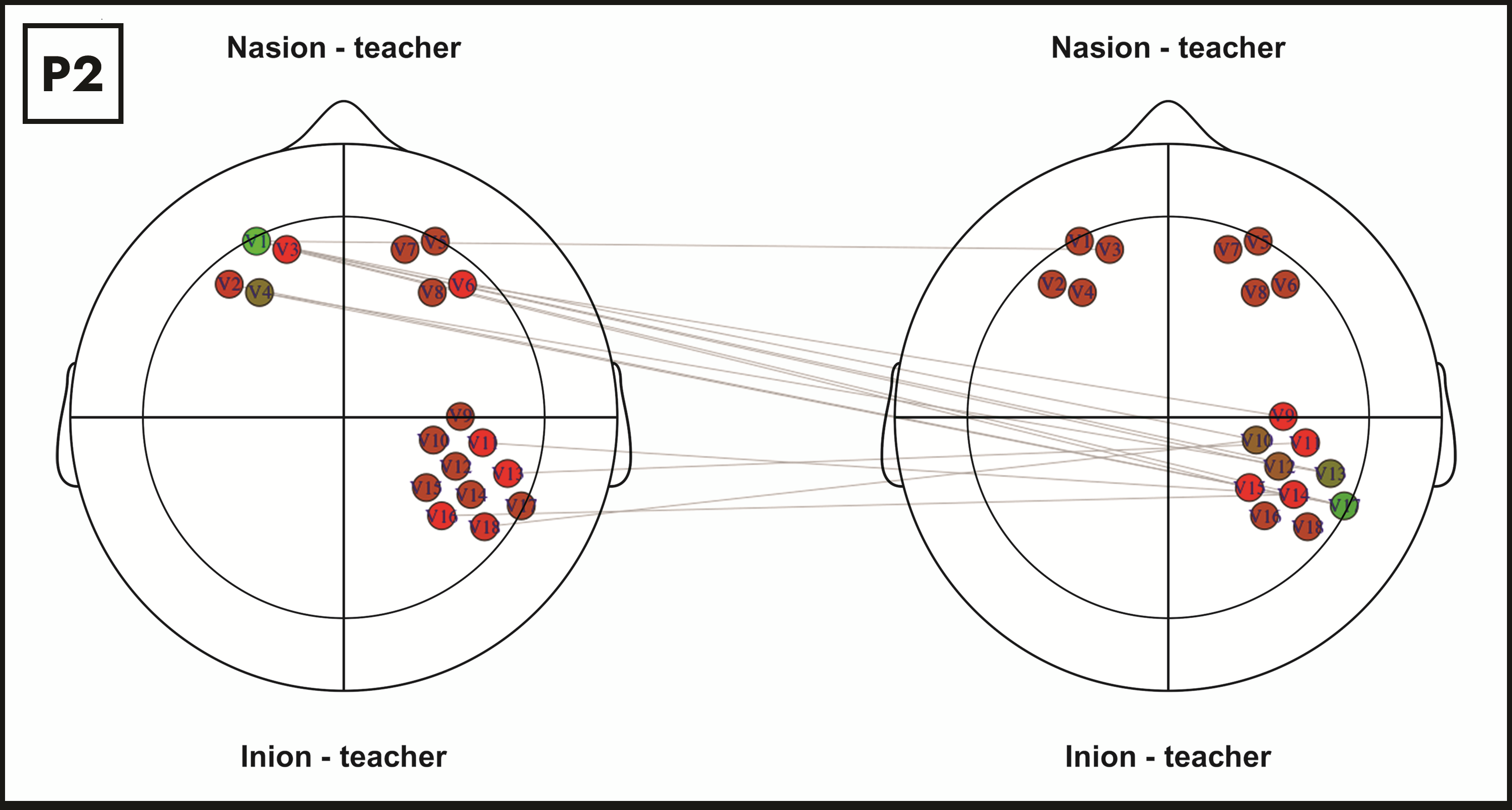

Supplement: Supplementary file 2 [file Image_2.TIF]
